# Clinical Significance of Severe and Borderline Umbilical Acidemia: Arterial pH, Lactate, Base Excess and Apgar Score Dynamics in Early Neonatal Risk Stratification

**DOI:** 10.3390/diagnostics16213562

**Published:** 2026-10-29

**Authors:** Beata Vida, Csongor Csetri-Barabasi, Janos Lenart, Zsuzsanna Molnar, Szabolcs Molnar, Tamas Deli, Zoard T. Krasznai

**Affiliations:** 1 Department of Obstetrics and Gynecology, Faculty of Medicine, University of Debrecen, 4032 Debrecen, Hungary; vida.beata@med.unideb.hu (B.V.); csetri.barabasi.csongor@med.unideb.hu (C.C.-B.); molnar.zsuzsanna@med.unideb.hu (Z.M.); molnar.szabolcs@med.unideb.hu (S.M.);; 2 Emergency Care Unit, Jahn Ferenc Dél-Pesti Kórház és Rendelőintézet, 1204 Budapest, Hungary; lenart.janos0819@gmail.com

**Keywords:** acidemia, Apgar, base excess, cord blood analysis, hypoxia, metabolic burden score, lactate, perinatal outcome, pH, pregnancy

## Abstract

**Background:** While Apgar score is still the cornerstone of perinatal assessment, umbilical cord blood acid–base analysis provides objective information on fetal acid–base status at birth. Guidelines recommend cord blood gas evaluation in selected high-risk cases of low Apgar scores. However, newborns with severe and borderline acidemia may have temporarily reassuring Apgar scores despite biochemical evidence of metabolic stress. This study aimed to evaluate the clinical significance of severe and borderline umbilical acidemia, especially in the case of pH 7.19–7.10 values as a gray zone, and to assess whether lactate, base excess and metabolic burden score improve neonatal risk stratification. **Methods:** We performed a retrospective analysis of 1.989 newborns with available (routine) umbilical cord arterial blood gas parameters and 1 and 5 min Apgar scores. Severe and borderline acidemia were defined as arterial pH < 7.10 and 7.19–7.10, respectively. Arterial pH, lactate and base excess parameters were assessed, along with 1 and 5 min Apgar scores and early perinatal outcomes including the need for neonatal intensive care unit admission and CPAP requirement. **Results:** Close observation or NICU admission was associated with lower pH, higher lactate, and more negative base excess with a higher metabolic burden score. Among newborns with arterial pH < 7.20 (*n* = 299), ROC analysis showed lactate with the highest discriminative performance for close observation or NICU admission (AUC 0.783), followed by metabolic burden score (AUC 0.759). Both lactate and the metabolic burden score outperformed the pH risk alone. Although Apgar scores were statistically lower among admitted newborns, median 5 min Apgar values remained clinically reassuring. **Conclusions:** Severe and borderline umbilical arterial acidemia may identify neonates requiring closer observation. Integrating lactate, base excess, metabolic burden score and Apgar score dynamics may improve early risk stratification to discover perinatal hypoxia, with identification of newborns requiring close postnatal observation.

## 1. Introduction

Perinatal hypoxia remains one of the most relevant challenges in obstetric and neonatal care; delayed diagnosis may contribute to early neonatal morbidity in severe cases and long-term neurological conditions. At birth, the clinical condition of the newborn is routinely assessed by the Apgar score, but this scoring system primarily reflects early signs of adaptation and resuscitation need; therefore, in the case of temporarily reassuring Apgar scores, the need for close monitoring is not necessarily recognized [1,2,3].

There is a very important forensic aspect of umbilical blood gas analysis. In the event of cerebral palsy, according to the American College of Obstetricians and Gynecologists, there is a threshold of ≥12 mmol/L base deficit (BD), or a pH < 7.0 as a criterion to be consistent with an acute peripartum or intrapartum asphyxia event to support the diagnosis. This makes umbilical cord blood analysis essential not only as quality control but also to avoid litigation [4].

Umbilical cord blood gas analysis provides an objective assessment of neonatal metabolic adaptation: arterial pH shows the hydrogen ion concentration, and base excess (BE) helps to characterize the metabolic component of acidemia, distinguishing metabolic and respiratory acidosis. An elevated lactate level is direct evidence of anaerobic metabolism. Although these metabolic values can undergo a strong alteration in the case of severe acidosis, they should not be assessed in isolation but could be integrated, collectively measuring a complex adaptation failure at birth [1].

The importance of severe acidemia is emphasized, defined as a pH below 7.10 in blood drawn from an umbilical arterial blood sample; nevertheless, clinical interpretation of borderline acidemia remains unclear. Newborns with pH values above the classical severe acidemia can also demonstrate short-term complications and the need for resuscitation with close observation or even neonatal intensive care unit (NICU) admission. It has been shown in large cohorts that the relationship between umbilical cord arterial pH and severe adverse neonatal outcomes is not restricted to a single cut-off value, and an increased risk for complications may be experienced close to pH 7.10 [5].

Other population-based data showed increased neonatal morbidity and mortality for newborns with pH values between 7.00 and 7.19 compared to those with pH values above 7.20, which can necessitate the reconsideration of close observation of newborns and indication of admission for NICU [6]. Consequently, pH values between 7.10 and 7.19 can represent a clinically relevant “gray zone”. This range is not traditionally defined as severe acidemia, but it might suggest a transitional state of metabolic stress. In this subgroup, infants may have apparently acceptable Apgar scores while umbilical cord blood gas parameters can predict the anaerobic metabolic load. The discrepancy between clinical adaptation and biochemical status needs to be seriously handled because using Apgar scores alone can lead to under-recognition, postponing early surveillance [7,8].

Several studies support the concept that umbilical cord arterial pH with additional metabolic markers such as lactate and base excess could be integrated in a complex evaluation form. Since lactate is a direct biochemical proof of anaerobic metabolism, and therefore is elevated in intrapartum asphyxia, sometimes its value is more discriminating than arterial pH in predicting neonatal morbidity [9,10,11]. In the identification of perinatal tissue, hypoxia pH and lactate analysis seem to be equally effective [12]. Wiberg et al. examined the relationship between umbilical cord blood pH, base excess, lactate, 5 min Apgar score, and hypoxic–ischemic encephalopathy, confirming the need of comprehensive assessment of infants using metabolic markers and clinical condition evaluation [13]. The relationship between Apgar score and metabolic markers is also complex: a low Apgar score can occur without metabolic acidosis due to transient respiratory distress or prematurity, drug exposure, or adaptation delay. On the other hand, an acceptable 5 min Apgar score might occur with decreased pH and/or elevated lactate and base excess, suggesting metabolic stress. Therefore, identification of these newborns is important to avoid late detection-caused complications [14,15,16]. There might also be an inter-observer variability in the case of Apgar score, while pH and metabolic markers are objective. In a Swiss observational study, the Apgar scores determined by obstetricians were significantly lower than those determined by midwives or neonatologists [17].

The importance of early recognition of borderline umbilical acidemia warrants highlighting for several reasons. Focusing on newborns with pH 7.19–7.10 can help timely detection of increasing respiratory distress, feeding difficulties, neurologic alterations, seizures or signs of hypoxic–ischemic encephalopathy. With early identification, additional blood tests, glucose monitoring, neurologic evaluation or admission for NICU can be performed. The recognition of this gray zone may improve the communication between obstetricians and neonatologists, supporting risk-stratified early perinatal pathways.

This retrospective single-center study was performed to evaluate the clinical significance of borderline umbilical acidemia in a large cohort of 1.989 neonates, with the goal of refining neonatal risk stratification in perinatal hypoxia. We aimed to assess the relationship between umbilical cord arterial pH, lactate, base excess and 1 and 5 min Apgar score dynamics, especially in the case of pH < 7.10 and 7.19–7.10 as a gray zone. We also examined the rate of close observation and admission for NICU in severe and borderline acidemia. Evaluating individual acid–base parameters, combined interpretation of arterial pH, lactate, and base excess may provide clinically relevant representation of neonatal metabolic status. Previous studies have shown that low arterial pH alone is associated with adverse neonatal and long-term outcomes, but other biochemical markers should also be evaluated and interpreted together as a metabolic profile rather than being handled as isolated variables [13]. This study also examined the concept of metabolic burden score, defined as the number of abnormal acid–base parameters. A metabolic high-risk profile is defined as the co-existence of at least two abnormal biochemical markers. This approach aims to identify newborns with metabolic stress phenotype and characterize clinical-biochemical discordance observed in newborns who appear to be well adapted postnatally.

## 2. Materials and Methods

### 2.1. Study Design and Population

This retrospective single-center cohort study was performed at the tertiary-level institute of the University of Debrecen, Faculty of Medicine, Department of Obstetrics and Gynecology, Debrecen, Hungary. In the department, umbilical cord blood gas analysis has been routinely performed for quality assurance and medico-legal purposes since June 2021. Maternal, obstetric, and neonatal data were retrospectively collected between 1 January 2022 and 30 November 2022.

A total of 1.989 newborns were enrolled in the study. Eligibility criteria were singleton pregnancy, at least 37 weeks of gestation, technically valid and available umbilical cord arterial blood gas parameters (pH, lactate, base excess), and 1 and 5 min Apgar scores validated by neonatologists. Cases with preterm birth, multiple pregnancy, major structural and/or chromosomal abnormalities, fetal growth restriction, known placental insufficiency and altered fetal Doppler parameters, or incomplete umbilical cord blood gas analysis were excluded. The study was conducted in a tertiary obstetric referral center with an on-site level III perinatal intensive care unit providing neonatal intensive care.

### 2.2. Data Collection

Maternal and obstetric variables including gravidity, parity, gestational age at delivery, mode of delivery and maternal risk factors including obesity, diabetes, hypertensive disorders and smoking, were evaluated. Maternal obesity, gestational diabetes mellitus, and pregnancy-induced hypertension were selected as clinically relevant maternal comorbidities available in the dataset. Neonatal variables included birth weight, 1 and 5 min Apgar scores, and umbilical cord arterial blood gas parameters, such as pH, lactate, and base excess. Apgar score dynamics were assessed by comparing 1 and 5 min Apgar scores; the absolute change between the two values was used as an indicator of early neonatal adaptation. For clinical and biochemical discrepancy, exploratory subgroup analyses were performed. Metabolic burden score was developed as an exploratory study-specific variable reflecting the biochemical burden of neonatal metabolic stress. This marker quantifies the accumulation of abnormal biochemical parameters within the umbilical arterial pH–lactate–base excess axis. One point was assigned for each abnormal parameter: pH ≤ 7.10, lactate ≥ 8 mmol/L, base excess ≤ −12 mmol/L. Accordingly, the range scored from 0 to 3, where 0 was associated with the absence of predefined biochemical abnormalities, and 3 indicated concurrent abnormality of all three parameters. Metabolic high-risk profile was defined as the presence of at least two abnormal biochemical parameters. Apgar burden was defined as the difference between 1 and 5 min Apgar scores from the maximum value of 10. This variable was used only as an exploratory continuous measure of overall Apgar score reduction.

### 2.3. Umbilical Cord Arterial Blood Gas Analysis

After delivery, the umbilical cord was clamped, and an arterial blood sample was collected from an isolated cord segment, avoiding preanalytical variations. Arterial blood gas analysis was performed immediately (within 5 min) using a calibrated ABL90 FLEX PLUS blood gas analyzer (Radiometer Medical ApS, Bronshoj, Denmark) at the delivery room, Department of Obstetrics and Gynecology, University of Debrecen. For further analysis, arterial pH, lactate and base excess levels were selected as biochemical variables (pH as a marker of fetal acid–base status, base excess to evaluate the metabolic component of acidosis, and lactate as a biochemical marker of anaerobic metabolism related to impaired oxygen availability).

### 2.4. Definition of pH Subgroups

Based on the literature, pH categories were defined as follows, according to published reference ranges and clinically relevant thresholds [1,5,6]:

1. pH > 7.35 was the high-normal reference range.
2. pH 7.35–7.26 was the physiological/reference range.
3. pH 7.25–7.20 belonged to the low-normal reference group.
4. pH 7.19–7.10 belonged to borderline acidemia.
5. pH < 7.10 severe acidemia.

### 2.5. Outcomes

The primary endpoint was an early adverse neonatal outcome, defined as the need for close observation or NICU admission. Decisions regarding close observation or NICU admission were made according to institutional neonatal practice, including clinical condition at birth, respiratory adaptation, oxygen saturation, tone, feeding, and cord blood gas results, when available. Clinicians were not blinded to cord blood gas parameters, because these values were part of routine postnatal clinical assessment and neonatal handover. Close observation was defined as a need for structured postnatal monitoring in the neonatal unit or delivery room, including repeated clinical assessment of respiratory status, tone, feeding, oxygen saturation, and general adaptation without immediate need for intensive care treatment. As an additional neonatal outcome variable, the need for respiratory support was evaluated. Secondary endpoints were the discrepancy between arterial pH and 1 and 5 min Apgar scores, and the association between pH subgroups and lactate and base excess.

### 2.6. Statistical Analysis

The normality of variable distribution was assessed using the Shapiro–Wilk test. Association between arterial pH, lactate, base excess, and Apgar scores was evaluated using correlation analyses. Spearman’s rank correlation was used in the case of non-normally distributed variables. Independent group comparisons were tested using the Mann–Whitney U test; categorical variables were compared using Fisher’s exact test. Multivariable regression models were performed using Firth logistic regression. ROC and multivariable regression analyses were restricted to newborns with arterial pH < 7.20 (*n* = 299); PPV and NPV estimates were preselected for the low-pH subgroup. Because of the limited number of outcome events, multivariable regression was used just for the primary endpoint. Lactate and metabolic burden score were evaluated in separate models. Multivariable regression was not performed for CPAP because of the limited number of events and CPAP analyses were considered exploratory. A *p*-value < 0.05 was considered statistically significant.

Generative artificial intelligence (GenAI, Grammarly pro /v1.2.307.1989/) has been used in this paper for superficial text editing (grammar).

## 3. Results

### 3.1. Baseline Maternal, Obstetric and Neonatal Characteristics

Our results are presented in the following tables and figures, with Supplementary Information provided in Supplementary File Tables S1–S7, Figure S1 and Figure S2. The baseline maternal, obstetric, and neonatal characteristics of the 1.989 enrolled newborns and their mothers are summarized in Table 1. The study population consisted of term singleton newborns with technically valid umbilical arterial cord blood gas parameters and available 1 and 5 min Apgar scores. Maternal risk factors, including obesity, diabetes, hypertensive disorders, and smoking, as well as delivery mode, gestational age at birth, and neonatal birth weight, were recorded to characterize the clinical profile of the cohort. 

### 3.2. Distribution of Umbilical Arterial Acid–Base Parameters

A total of 1.989 newborns were enrolled in the analysis with available umbilical cord blood gas parameters and Apgar scores; after the Shapiro–Wilk test for further analyses, non-parametric statistical methods were used (Table 2).

### 3.3. Association Between Arterial pH, Lactate, Base Excess

Severe and borderline acidemia subgroups were defined as umbilical arterial pH < 7.20, differentiating them with the value of 7.10. pH, lactate, and base excess were strongly correlated, supporting the interpretation of these parameters as common metabolic stress components (Table 3).

### 3.4. Biochemical Markers and Apgar Score Dynamics

**Figure 1:**
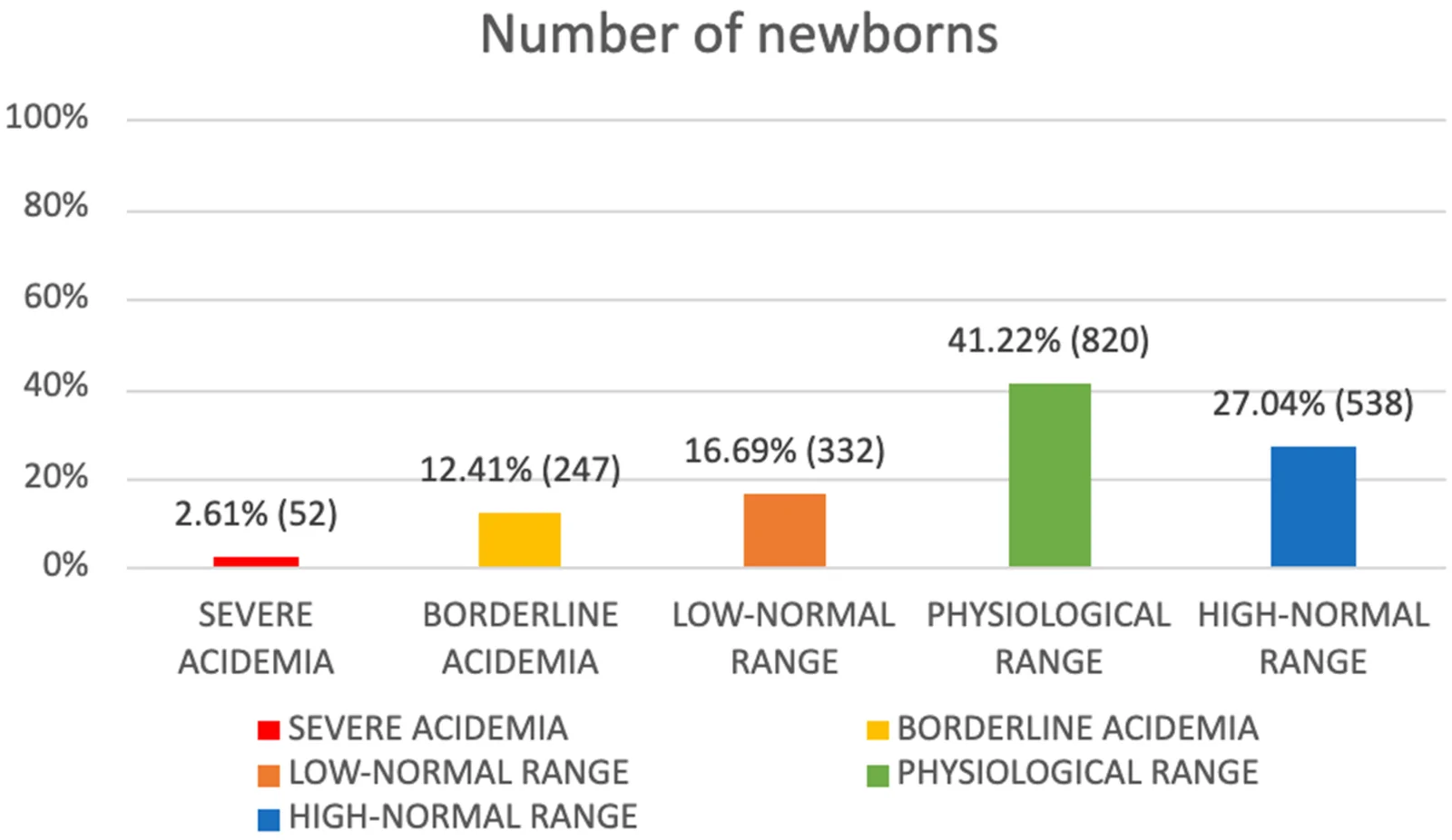
Distribution of umbilical arterial pH subgroups.

Newborns were categorized in predefined subgroups to evaluate whether pH ranges were associated with differences in Apgar score dynamics. The distribution of patients in pH subgroups was in accordance with those of other tertiary institutions [6]. The distribution of subgroups was as follows: 27.04% high–normal range, 41.22% physiological range, 16.69% low–normal range, 12.41% borderline acidemia, and 2.61% severe acidemia, as shown in Figure 1.

The subgroup of acidemia (pH < 7.20) was classified further as borderline (pH 7.19–7.10) and severe (pH < 7.10) acidemia, with further subgroup analyses below this range. Comparing acid–base parameters with Apgar 1 and 5 min scores, the degree of metabolic variability was not reliably reflected (Table 4).

### 3.5. Close Observation or Neonatal Intensive Care Unit Admission in Severe and Borderline Acidemia

Among newborns with umbilical arterial pH < 7.20, 52 infants were classified in the severe acidemia subgroup and 247 in the borderline acidemia subgroup. Overall, 31 of these 299 patients (10.4%) required close observation or NICU admission; 8/52 patients (15.4%) from the severe and 23/247 (9.3%) from the borderline acidemia subgroup. CPAP requirement was significantly more frequent in the subgroup of severe acidemia (6/52 vs. 6/247 newborns) (Table 5).

Although Apgar scores were statistically lower among newborns who required close observation or NICU admission, median 1 and 5 min values remained clinically reassuring. The median 5 min Apgar score was 10 in both subgroups, and the overall distribution was significantly lower among newborns requiring close observation or NICU admission (Mann–Whitney U test *p* < 0.001). Apgar 5 min < 9 was significantly more frequent among admitted newborns than those managed with standard care (8/268 3.0% vs. 9/31 29.0%, Fisher’s exact test *p* < 0.001). However, Apgar 5 min < 7 was not significantly associated with close observation or NICU admission (Fisher’s exact test *p* = 0.356). The data suggest that seemingly favorable early clinical adaptation may co-exist with biochemical metabolic stress.

### 3.6. Metabolic Burden Score and Metabolic High-Risk Profile

Further exploratory subgroup analyses were performed to evaluate the predictive value of combined biochemical markers related to metabolic stress: the metabolic burden score was significantly higher in cases with close observation or NICU admission (Table 6).

A lactate threshold ≥ 8 mmol/L was strongly associated with close observation or NICU admission and was present in 22/31 newborns (71.0%) (Fisher’s exact test *p* < 0.001). More pronounced metabolic acidosis reflected by a BE threshold ≤ −12 mmol/L was more frequent among admitted newborns, although this biomarker was less sensitive than lactate (Fisher’s exact test *p* = 0.039).

**Figure 2:**
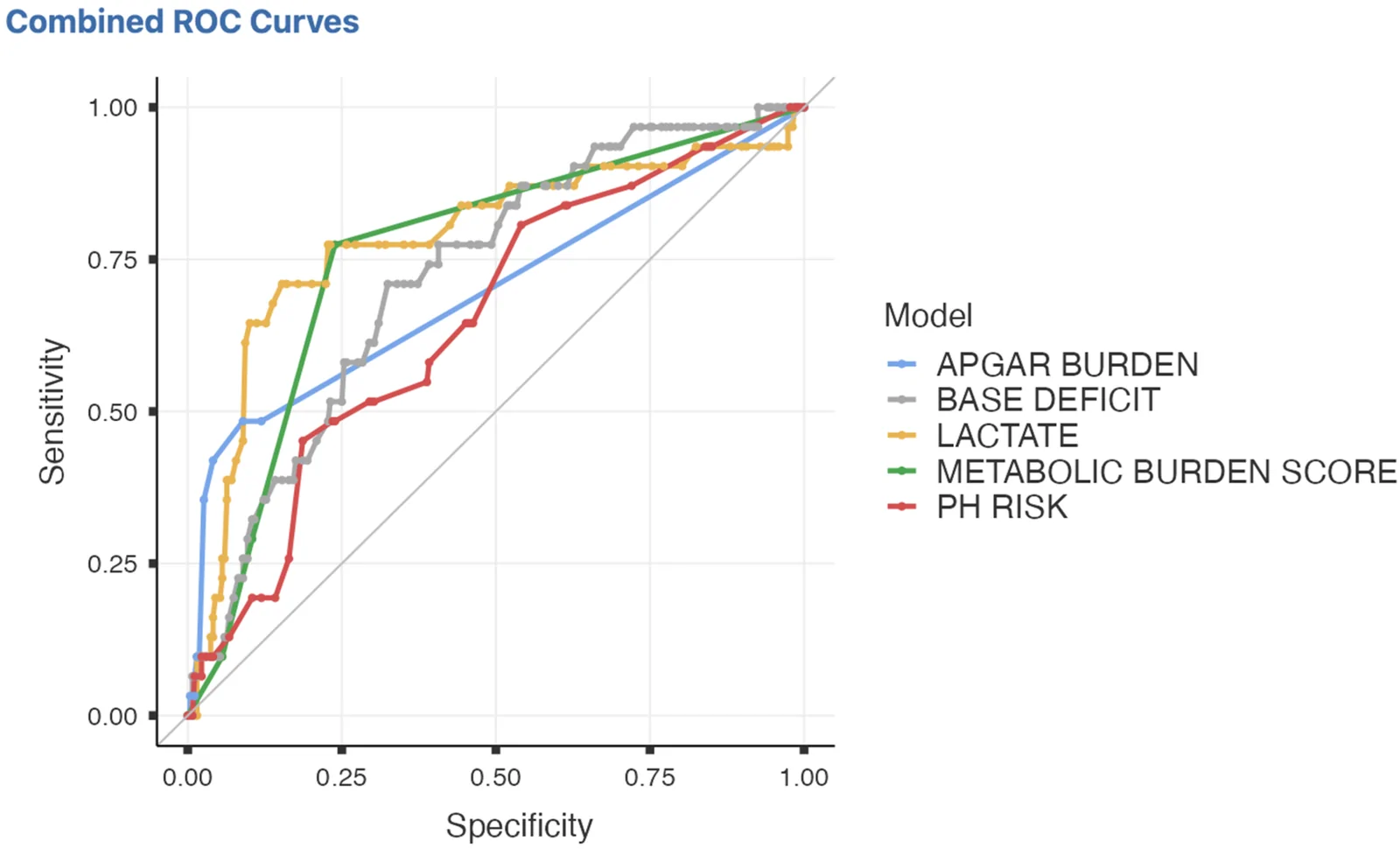
Combined ROC analysis.

In Receiver Operating Characteristic (ROC) analysis, lactate showed the highest discriminative performance followed by the metabolic burden score. The optimal lactate cut-off value defined by the Youden index was 8.05 mmol/L, close to the predefined lactate threshold (Table 7, Figure 2). pH risk was calculated as a deviation below the predefined threshold of 7.20, with higher values indicating greater biochemical alterations; this value alone was less sensitive than other variables.

Pairwise AUC comparisons using DeLong’s test demonstrated better discriminative performance of the metabolic burden score than pH risk alone (AUC difference: 0.108, 95% CI: 0.027–0.189, *p* = 0.009). Lactate also outperformed pH risk (AUC difference: 0.132, 95% CI: 0.042–0.223, *p* = 0.004). No significant difference was observed between lactate and the metabolic burden score (*p* = 0.441). Apgar burden, calculated as a cumulative deviation of 1 and 5 min Apgar scores from the ideal value of 10, was significantly higher among infants requiring close observation or NICU admission, although its discriminative performance was lower than that of lactate and metabolic burden.

Metabolic high-risk profile, defined by the presence of at least two abnormal biochemical parameters, was observed only in 9 of 31 newborns (29.0%) associated with close observation or NICU admission, suggesting that the accumulation of biochemical abnormalities may identify a subgroup with increased clinical variability (Fisher’s exact test *p* = 0.007).

### 3.7. Biochemical Predictors of Early Neonatal Outcomes

In a multivariable logistic regression model, lactate remained independently associated with close observation or NICU admission after adjustment for maternal obesity, gestational diabetes, and pregnancy-induced hypertension (OR 1.66 per 1 mmol/L increase, 95% CI 1.35–2.04, *p* < 0.001). In a separate adjusted model, each one-point increase in metabolic burden score was associated with higher odds of close observation or NICU admission (OR 2.12, 95% CI 1.50–3.01, *p* < 0.001). Given the limited number of CPAP events (*n* = 12), CPAP analyses were considered exploratory and were not subjected to multivariable adjustment.

## 4. Discussion

The analysis of the results of our retrospective cohort of 1.989 newborns confirmed a weak correlation between the biochemical acid–base parameters and Apgar scores in newborns with severe or borderline acidemia. This suggests that metabolic stress is not necessarily reflected in early clinical adaptation scores and supports the concept that biochemical parameters, especially pH, lactate, and base excess, reflect real aspects of fetal acid–base status at birth. Our data also support previous evidence emphasizing the clinical utility of umbilical arterial blood gas analysis in assessing early neonatal condition [18,19,20]. Olofsson et al. highlighted that blood gas parameters should not be interpreted as isolated values, but rather as complementary components of fetal oxygenation, acid–base balance, and metabolic adaptation at birth [1]. Armstrong et al. emphasized the relevance of cord blood gas analysis as an objective measure of neonatal condition, given that several non-metabolic factors influence Apgar scores [3]. Although current ACOG and NICE recommendations support selective cord blood gas analysis mainly in newborns in poor condition or after high-risk deliveries, our findings suggest that biochemical metabolic stress may also be present despite reassuring Apgar scores, supporting the potential value of broader acid–base assessment in selective tertiary-care settings [15,21]. On the contrary, the guideline of the Society of Obstetricians and Gynecologists of Canada recommends universal paired (arterial and venous) umbilical cord blood gas testing. The latter recommendation explains the reason for universal testing. This, besides providing appropriate care for the newborn, is useful for quality assurance and quality improvement as well. There is very limited evidence that universal cord blood gas testing has benefits compared to selected testing in terms of neonatal outcome, and the study focused on severe acidemia (pH < 7.10). A single-center study supported the cost-effectiveness of universal testing, but because the findings were limited to one medical center, the evidence remains insufficient and needs to be supported by further results [7,22,23,24]. In our medical center and financial setting, the costs of selected and universal testing would be the same, due to the relatively lower price of larger sets of reagents and the limited expiration time of the kits.

While severe acidemia has traditionally received the greatest attention, our data suggest that borderline acidemia may also represent a clinically meaningful subgroup when biochemical abnormalities co-exist with reassuring Apgar scores. This interpretation is supported by large international cohorts demonstrating that decreasing arterial cord pH is associated with adverse neonatal outcomes [25,26,27]. Yeh et al., including 51.519 validated samples, reported serious adverse neonatal outcomes with decreasing arterial cord pH and an increase in clinically relevant risk around pH close to 7.10 [5]. Andersson et al. reported increased neonatal morbidity and mortality not only in cases of pH below 7.00, but in those newborns with pH levels between 7.00 and 7.19 as well, supporting the evidence of borderline acidemia [6]. Bailey et al. similarly presented that borderline neonatal acidemia at term was associated with increased short-term neonatal morbidity [8]. These findings provide a strong rationale for close observation of those in the severe and borderline pH categories. A systematic review and meta-analysis by Malin et al. established a strong correlation between low arterial cord pH and hypoxic–ischemic encephalopathy, intraventricular hemorrhage, periventricular leukomalacia, and cerebral palsy [2,28]. Within the low pH subgroup, arterial pH, lactate, and base excess showed no significant correlations with either 1 or 5 min Apgar scores. These findings suggest that the degree of biochemical metabolic disturbance was not directly reflected by early Apgar scores in this subgroup. Importantly, the lack of correlation with Apgar scores should be distinguished from the predictive performance of lactate for subsequent clinical management outcomes, which was demonstrated by the ROC and multivariable regression analyses. Lactate is more directly related to anaerobic metabolism during impaired oxygen availability [29,30]. Tuuli et al. reported a stronger predictive value of umbilical cord arterial lactate in neonatal morbidity at term [9]. Allanson et al. also supported the relevance of lactate as a predictor of neonatal risk [11]. Wiberg et al. evaluated the umbilical cord arterial pH, lactate, base deficit, and 5 min Apgar score in relation to hypoxic–ischemic encephalopathy, demonstrating the importance of integrated biochemical and clinical assessment [13]. In our cohort, lactate showed weak negative, but non-significant correlations with both 1 min and 5 min Apgar scores. Base excess provides additional information on the metabolic component of acidemia; however, no significant correlation with Apgar scores was observed. In subgroup analysis, base excess was more negative among newborns requiring close observation or NICU admission. Previous studies have shown that base excess may help to identify newborns with an increased risk of complications [31,32]. Gover et al. suggested that base excess is an important value in risk stratification of short-term clinical outcomes in cases of severe and borderline acidemia [33].

One of the most relevant findings of our study is the discrepancy between Apgar score-based clinical assessment and biochemical alterations signifying metabolic stress. Subgroup analysis with severe and borderline acidemia identified 31 newborns who required close observation or NICU admission. Although 5 min Apgar scores were statistically lower among admitted newborns, median 5 min Apgar values remained reassuring, and only one admitted infant had a 5 min Apgar score below 7. This suggests that conventional Apgar-based assessment alone may underestimate metabolic variability in this subgroup [34]. Since alteration from the Apgar score of 10 (e.g., a 5 min Apgar score of 9) can be considered clinically reassuring, Apgar burden should be interpreted as a mathematical descriptor of score distribution rather than a diagnostic marker of abnormal neonatal adaptation. Sabol and Caughey demonstrated the presence of acidemia with a 5 min Apgar score of 7 or greater; previous study results showed further discrepancy between the Apgar score and umbilical cord acid–base status [27,35,36,37]. Mlodawska et al. reported statistically significant but weak correlations between Apgar scores and umbilical cord venous pH and bicarbonate levels in term and late preterm newborns born in medium and severe conditions; therefore, clinical and biochemical assessments should be considered complementary [38]. International data point out that favorable postnatal adaptation should not automatically exclude metabolic vulnerability.

Identifying clinical and biochemical discordance, we explored the concepts of metabolic burden score and metabolic high-risk profile. We defined metabolic burden score as the number of abnormal biochemical parameters on the pH–lactate–base excess axis, and metabolic high-risk profile as the presence of at least two abnormal biochemical markers. These parameters are not proposed as validated diagnostic scores, but they can provide clinically useful interpretation by combining biochemical abnormalities [39]. Our subgroup analysis showed that the metabolic burden score was higher among newborns requiring close observation or NICU admission (*p* = <0.001), while metabolic high-risk profile was present in 9 of 31 newborns (29.0%, *p* = 0.007). Lactate ≥ 8 mmol/L was observed in 22 of 31 newborns (71.0%); it showed the strongest discriminative performance for close observation or NICU admission. These results support the hypothesis that a combined biochemical profile may identify newborns with metabolic instability. The lactate values and metabolic burden score showed the largest discrepancies among the evaluated variables, although this result should be interpreted cautiously. The DeLong’s pairwise AUC comparisons further support the concept that umbilical arterial pH alone may be insufficient for early risk stratification, but lactate and the metabolic burden score showed significantly higher discriminative performance than pH alone. Of the investigated parameters, lactate had the highest AUC, but the metabolic burden score did not differ significantly from it. The metabolic burden score should not be interpreted as being superior to lactate, but rather as an exploratory framework for summarizing concurrent biochemical abnormalities. Lactate may capture clinically relevant metabolic stress more effectively than pH alone. Combining pH, lactate, and base excess into an integrated metabolic profile may provide additional context for identifying newborns requiring close postnatal observation.

Vesoulis et al. reconsidered arterial cord pH as not being a stand-alone screening criterion for neonatal encephalopathy around pH 7.10 [7]. Our results have practical implications for neonatal risk stratification, aiming to identify a subgroup in whom close observation or NICU admission may be justified while avoiding overmedicalization of affected newborns. Newborns with positive metabolic burden scores and metabolic high-risk profiles can benefit from structured early monitoring, including repeated clinical assessment, respiratory observation, blood glucose surveillance, and neurologic surveillance when indicated [40]. This approach may help to detect respiratory distress, impaired adaptation, or early signs of hypoxic–ischemic encephalopathy before serious complications [28]. Focusing not only on severe but also borderline acidemia, this study addresses the clinically relevant gray zone in neonatal assessment, and subgroup analysis provides additional insight into the potential mismatch between clinical adaptation and biochemical stress.

Previous data also demonstrated that clinical neonatal assessment and umbilical cord blood gas parameters may not always change in parallel. The twisting of the umbilical cord around the fetal neck (nuchal cord) has been associated with lower 1 min Apgar scores without a corresponding increase in the frequency of low arterial umbilical cord pH [41]. These findings further support a complementary, rather than interchangeable, interpretation of these parameters. In the present study, we did not specifically evaluate the presence of the umbilical cord around the fetal neck or other individual obstetric conditions as potential determinants of discordance between Apgar scores and biochemical parameters, but the contribution of obstetric factors may be a relevant topic for future studies.

Limitations include that the results were obtained from a retrospective single-center study; therefore, our findings may be influenced by institutional sampling and admission rules. Close observation and NICU admission are practice-dependent outcomes and may not fully reflect objective neonatal morbidity, and the number of close observation or NICU admission events in severe and borderline subgroups was small; therefore, the statistical power is limited. Because clinicians were not blinded to the cord blood gas results, incorporation bias cannot be excluded, particularly for the endpoint of close observation or NICU admission. Therefore, the ROC results may reflect local neonatal management pathways rather than purely the biological prediction on neonatal morbidity. The metabolic burden score and metabolic high-risk profile require validation in larger independent cohorts. The metabolic burden score was developed and evaluated within the same dataset; therefore, its predictive performance may be affected by statistical overfitting. This exploratory score should be validated in an independent prospective cohort before clinical implementation. ROC-derived cut-offs were considered exploratory and were not intended as ready-to-use clinical decision thresholds. Long-term neurodevelopmental outcomes were not available due to the retrospective nature of the study; that is why this analysis focuses on early neonatal adaptation and short-term monitoring [42,43].

## 5. Conclusions

In conclusion, our findings support the clinical relevance of integrating umbilical arterial pH, lactate, and base excess with Apgar score dynamics in the early assessment of newborns affected by perinatal hypoxia. Severe and borderline umbilical arterial acidemia may identify newborns who require close postnatal assessment when accompanied by elevated lactate and negative base excess. Lactate showed the strongest discriminatory performance for close observation or NICU admission. At the same time, the metabolic burden score provided an exploratory summary of concurrent biochemical abnormalities but did not outperform lactate alone. Reassuring Apgar scores may not fully exclude biochemical metabolic stress. The results also suggest that combined interpretation of biochemical markers may improve early neonatal risk stratification and help identify infants who may benefit from close observation or NICU admission in the postnatal period. Because the number of events was limited and the thresholds were derived from the same cohort, these findings should be interpreted as exploratory and require validation in independent prospective studies.

## Figures and Tables

**Table 1:** Baseline maternal, obstetric, and neonatal characteristics of the study cohort. Values are presented as median (IQR) or *n* (%).

Variable	Total Cohort, *n* = 1.989
Maternal BMI, kg/m^2^, median	29.3 (26.1–32.8)
Gestational age at delivery (weeks), median	39.4 (38.6–40.2)
Vaginal delivery, *n* (%)	1343 (67.6%)
Cesarean delivery, *n* (%)	568 (28.6%)
Operative vaginal delivery, *n* (%)	76 (3.8%)
Maternal obesity (BMI ≥ 30 kg/m^2^), *n* (%)	865 (43.6%)
Diabetes, *n* (%)	129 (6.5%)
Hypertensive disorder, *n* (%)	128 (6.5%)
Smoking, *n* (%)	233 (11.8%)
1 min Apgar score, median	9
5 min Apgar score, median	10
Arterial pH, median	7.30 (7.24–7.35)
Lactate, mmol/L, median	3.40 (2.20–4.80)
Base excess, mmol/L, median	−3.00 (−5.40–0.06)
pH ≤ 7.20, *n* (%)	299 (15%)
Close observation or NICU admission, *n* (%)	31 (1.6%)
CPAP requirement, *n* (%)	12 (0.6%)

**Table 2:** Distribution of umbilical arterial acid–base parameters.

Parameter	Median	IQR	Min–Max	Shapiro–Wilk W	*p*-Value
pH	7.30	(7.24–7.35)	6.82–7.60	0.976	*p* < 0.001
lactate (mmol/L)	3.4	(2.20–4.80)	0.80–15.00	0.924	*p* < 0.001
base excess (mmol/L)	−3.00	(−5.40–−0.060)	19.60–24.00	0.977	*p* < 0.001

**Table 3:** Association between acid–base parameters in low pH subgroups.

Correlation	Spearman’s r	*p*-Value
pH vs. lactate	−0.608	*p* < 0.001
pH vs. base excess	0.469	*p* < 0.001
lactate vs. base excess	−0.684	*p* < 0.001

**Table 4:** Association between acid–base parameters and Apgar scores in the subgroup of acidemia (pH < 7.20).

Parameter vs. Apgar	Spearman’s r	*p*-Value
pH vs. Apgar 1	0.070	0.227
pH vs. Apgar 5	0.062	0.287
lactate vs. Apgar 1	−0.074	0.202
lactate vs. Apgar 5	−0.078	0.176
BE vs. Apgar 1	0.007	0.906
BE vs. Apgar 5	0.006	0.918

**Table 5:** Severe vs. borderline acidemia: close observation or NICU admission and CPAP.

pH Subgroup	Close Observation or NICU Admission *n* (%)	CPAP Requirement *n* (%)
severe acidemia (pH < 7.10)	8/52 (15.4%)	6/52 (11.5%)
borderline acidemia (pH 7.19–7.10)	23/247 (9.3%)	6/247 (2.4%)
	*p* = 0.192 ^×^	*p* = 0.008 ^××^

× Pearson’s X^2^ test, ×× Fisher’s exact test.

**Table 6:** Median of biochemical parameters and metabolic burden score in case of standard care compared with close observation or NICU admission (low pH subgroup).

Variable (Median)	Standard Care	Close Observation or NICU Admission	*p*-Value ^×^
pH	7.15	7.12	0.006
lactate (mmol/L)	6.30	8.60	<0.001
BE (mmol/L)	−7.70	−9.90	<0.001
metabolic burden score	0	1	<0.001

**^×^** Mann–Whitney U test.

**Table 7:** Exploratory ROC-derived thresholds associated with close observation or NICU admissions.

Variable (Cut-Off)	AUC (95% CI)	Sensitivity (95% CI)	Specificity (95% CI)	PPV (95% CI)	NPV (95% CI)	*p*-Value ^1^
pH risk (0.045)	0.651 (0.551–0.75)	80.65% (63.72–90.81)	45.90% (40.03–51.88)	14.71% (10.16–20.81)	95.35% (90.22–97.85)	0.003
Lactate (≥8.05 mmol/L)	0.783 (0.681–0.885)	70.97% (53.41–83.90)	84.70% (79.90–88.52)	34.94% (24.33–47.25)	96.19% (92.91–97.98)	<0.001
Base deficit (≥8.95 mmol/L)	0.721 (0.633–0.810)	70.97% (53.41–83.90)	67.54% (61.72–72.86)	20.18% (13.72–28.67)	95.26% (91.24–97.49)	<0.001
Metabolic burden score (≥0.5)	0.759 (0.678–0.840)	77.42% (60.19–88.60)	76.12% (70.67–80.83)	27.27% (19.07–37.38)	96.68% (93.31–98.38)	<0.001
Apgar burden (≥2.5)	0.698 (0.601–0.795)	48.39% (31.97–65.16)	91.04% (87.02–93.91)	38.46% (24.89–54.10)	93.85% (90.24–96.18)	<0.001

^1^ ROC analysis.

## Data Availability

The data presented in this study are available from the corresponding author upon reasonable request.

## Supplementary Materials

The following supporting information can be downloaded at https://www.mdpi.com/article/10.3390/diagnostics16213562/s1, Table S1: Baseline maternal, obstetric and neonatal characteristics of the study cohort. Values are presented as median (IQR) or *n* (%); Table S2: Distribution of umbilical arterial acid–base parameters; Table S3: Association between acid–base parameters in low pH subgroups; Table S4: Association between acid–base parameters and Apgar scores in the subgroup of acidemia (pH < 7.20); Table S5: Severe vs. borderline acidemia: Close observation or NICU admission and CPAP; Table S6: Median of biochemical parameters and metabolic burden score in case of standard care compared with close observation or NICU admission; Table S7: Exploratory ROC-derived thresholds associated with close observation or NICU admission; Figure S1: Distribution of umbilical arterial pH subgroups; Figure S2: Combined ROC analysis.

## Abbreviations

The following abbreviations are used in this manuscript:

AUC	Area under the curve
BE	Base excess
CPAP	Continuous positive airway pressure
IQR	Interquartile range
NICU	Neonatal intensive care unit
ROC	Receiver operating characteristic curve

## References

[1] Olofsson P. (2023). Umbilical cord pH, blood gases, and lactate at birth: Normal values, interpretation, and clinical utility. Am. J. Obstet. Gynecol..

[2] Malin G.L., Morris R.K., Khan K.S. (2010). Strength of association between umbilical cord pH and perinatal and long term outcomes: Systematic review and meta-analysis. BMJ.

[3] Armstrong L., Stenson B.J. (2007). Use of umbilical cord blood gas analysis in the assessment of the newborn. Arch. Dis. Child.-Fetal Neonatal Ed..

[4] Ross M.G. (2021). Forensic Analysis of Umbilical and Newborn Blood Gas Values for Infants at Risk of Cerebral Palsy. J. Clin. Med..

[5] Yeh P., Emary K., Impey L. (2012). The relationship between umbilical cord arterial pH and serious adverse neonatal outcome: Analysis of 51,519 consecutive validated samples. BJOG.

[6] Andersson C.B., Klingenberg C., Thellesen L., Johnsen S.P., Kesmodel U.S., Petersen J.P. (2024). Umbilical Cord pH Levels and Neonatal Morbidity and Mortality. JAMA Netw. Open..

[7] Vesoulis Z.A., Liao S.M., Rao R., Trivedi S.B., Cahill A.G., Mathur A.M. (2018). Re-examining the arterial cord blood gas pH screening criteria in neonatal encephalopathy. Arch. Dis. Child.-Fetal Neonatal Ed..

[8] Bailey E.J., Frolova A.I., López J.D., Raghuraman N., Macones G.A., Cahill A.G. (2021). Mild Neonatal Acidemia is Associated with Neonatal Morbidity at Term. Am. J. Perinatol..

[9] Tuuli M.G., Stout M.J., Shanks A., Odibo A.O., Macones G.A., Cahill A.G. (2014). Umbilical cord arterial lactate compared with pH for predicting neonatal morbidity at term. Obstet. Gynecol..

[10] Tuuli M.G., Stout M.J., Macones G.A., Cahill A.G. (2016). Umbilical Cord Venous Lactate for Predicting Arterial Lactic Acidemia and Neonatal Morbidity at Term. Obstet. Gynecol..

[11] Allanson E.R., Waqar T., White C., Tunçalp Ö., Dickinson J.E. (2017). Umbilical lactate as a measure of acidosis and predictor of neonatal risk: A systematic review. BJOG.

[12] Bohiltea R.E., Mihai B.M., Ducu I., Cioca A.M., Bohiltea A.T., Iordache A.M., Iordache S.M., Grigorescu C.E.A., Marinescu S. (2022). Current Innovative Methods of Fetal pH Monitoring-A Brief Review. Diagnostics.

[13] Wiberg N., Källén K., Herbst A., Olofsson P. (2010). Relation between umbilical cord blood pH, base deficit, lactate, 5-minute Apgar score and development of hypoxic ischemic encephalopathy. Acta Obstet. Gynecol. Scand..

[14] Sabol B.A., Caughey A.B. (2016). Acidemia in neonates with a 5-minute Apgar score of 7 or greater—What are the outcomes?. Am. J. Obstet. Gynecol..

[15] ACOG Committee on Obstetric Practice (2006). ACOG Committee Opinion No. 348, November 2006: Umbilical cord blood gas and acid-base analysis. Obstet. Gynecol..

[16] Committee on Obstetric Practice, American Academy of Pediatrics Committee on Fetus and Newborn (2015). The Apgar Score. Obstet. Gynecol..

[17] Arri S.J., Bucher H.U., Merlini M., Fauchère J.C. (2018). Inter-Observer Variability of the Apgar Score of Preterm Infants between Neonatologists, Obste-tricians and Midwives. J. Neonatol. Clin. Pediatr..

[18] Martí Gamboa S., Pascual Mancho J., Rodrigo Rodríguez M., Ruiz Sada J., Castán Mateo S. (2017). pH, base deficit or lactate. Which is better for predicting neonatal morbidity?. J. Matern. Fetal Neonatal Med..

[19] Neacsu A., Herghelegiu C.G., Voinea S., Dimitriu M.C., Ples L., Bohiltea R.E., Braila A.D., Nastase L., Bacalbasa N., Chivu L.I. (2021). Umbilical cord lactate compared with pH as predictors of intrapartum asphyxia. Exp. Ther. Med..

[20] Çetinkaya Demir B., Orhaner A. (2026). Association of Intrapartum Cardiotocography Findings with Umbilical Arterial Blood Gas Parameters and Neonatal Outcomes in Non-Reassuring Fetal Status. J. Clin. Med..

[21] National Institute for Health and Care Excellence (NICE) (2023). Intrapartum care. NICE Guideline.

[22] Liston R., Sawchuck D., Young D. (2018). No. 197b-Fetal Health Surveillance: Intrapartum Consensus Guideline. J. Obstet. Gynaecol. Can..

[23] Nichols J.H., Ali M., Anetor J.I., Chen L.S., Chen Y., Collins S., Das S., Devaraj S., Fu L., Karon B.S. (2022). AACC Guidance Document on the Use of Point-of-Care Testing in Fertility and Reproduction. J. Appl. Lab. Med..

[24] White C.R., Doherty D.A., Cannon J.W., Kohan R., Newnham J.P., Pennell C.E. (2016). Cost effectiveness of universal umbilical cord blood gas and lactate analysis in a tertiary level maternity unit. J. Perinat. Med..

[25] Lau S.L., Lok Z.L.Z., Hui S.Y.A., Fung G.P.G., Lam H.S., Leung T.Y. (2023). Neonatal outcome of infants with umbilical cord arterial pH less than 7. Acta Obstet. Gynecol. Scand..

[26] Williams K.P., Singh A. (2002). The correlation of seizures in newborn infants with significant acidosis at birth with umbilical artery cord gas values. Obstet. Gynecol..

[27] Josten B.E., Johnson T.R., Nelson J.P. (1987). Umbilical cord blood pH and Apgar scores as an index of neonatal health. Am. J. Obstet. Gynecol..

[28] Pedersen M.V., Lindhard M.S., Moster D., Lie R.T., Henriksen T.B. (2026). Cerebral Palsy Risk by Combined Apgar Score and Umbilical Cord Blood pH Levels. JAMA Netw. Open..

[29] Cahill A.G., Macones G.A., Smyser C.D., López J.D., Inder T.E., Mathur A.M. (2017). Umbilical Artery Lactate Correlates with Brain Lactate in Term Infants. Am. J. Perinatol..

[30] Van Anh T.N., Hao T.K., Chi N.T.D., Son N.H. (2019). Predictions of Hypoxic-Ischemic Encephalopathy by Umbilical Cord Blood Lactate in Newborns with Birth Asphyxia. Open Access Maced. J. Med. Sci..

[31] Victory R., Penava D., da Silva O., Natale R., Richardson B. (2003). Umbilical cord pH and base excess values in relation to neonatal morbidity for infants delivered preterm. Am. J. Obstet. Gynecol..

[32] Low J.A., Lindsay B.G., Derrick E.J. (1997). Threshold of metabolic acidosis associated with newborn complications. Am. J. Obstet. Gynecol..

[33] Gover A., Riskin A., Sharkansky L., Hijazi R., Ghannam R., Zaitoon H. (2025). Short-Term Clinical and Biochemical Outcomes of Infants Born After 34 Weeks of Gestation with Mild-to-Moderate Cord Blood Acidosis-A Retrospective Study. J. Clin. Med..

[34] Boos V., Bührer C. (2024). Trends in Apgar scores and umbilical artery pH: A population-based cohort study on 10,696,831 live births in Germany, 2008–2022. Eur. J. Pediatr..

[35] Helwig J.T., Parer J.T., Kilpatrick S.J., Laros R.K. (1996). Umbilical cord blood acid-base state: What is normal?. Am. J. Obstet. Gynecol..

[36] Boehm F.H., Fields L.M., Entman S.S., Vaughn W.K. (1986). Correlation of the one-minute Apgar score and umbilical cord acid-base status. South Med. J..

[37] Hoffmann A.L., Hjortdal J.O., Secher N.J., Weile B. (1991). The relationship between Apgar score, umbilical artery pH and operative delivery for fetal distress in 2778 infants born at term. Eur. J. Obstet. Gynecol. Reprod. Biol..

[38] Mlodawska M., Mlodawski J., Gladys-Jakubczyk A., Pazera G. (2022). Relationship between Apgar score and umbilical cord blood acid-base balance in full-term and late preterm newborns born in medium and severe conditions. Ginekol. Pol..

[39] Vanspranghels R., Houfflin-Debarge V., Deken V., Rakza T., Maboudou P., Storme L., Ghesquiere L., Garabedian C. (2020). Umbilical cord arterial and venous gases, ionogram, and glucose level for predicting neonatal morbidity at term. Eur. J. Obstet. Gynecol. Reprod. Biol..

[40] De Bernardo G., De Santis R., Giordano M., Sordino D., Buonocore G., Perrone S. (2020). Predict respiratory distress syndrome by umbilical cord blood gas analysis in newborns with reassuring Apgar score. Ital. J. Pediatr..

[41] Młodawska M., Młodawski J., Świercz G., Zieliński R. (2022). The Relationship between Nuchal Cord and Adverse Obstetric and Neonatal Outcomes: Retrospective Cohort Study. Pediatr. Rep..

[42] Zaigham M., Källén K., Sundberg T.M., Olofsson P. (2025). Long-term outcomes after neonatal acidemias. Am. J. Obstet. Gynecol..

[43] Leinonen E., Gissler M., Haataja L., Andersson S., Rahkonen P., Rahkonen L., Metsäranta M. (2019). Umbilical artery pH and base excess at birth are poor predictors of neurodevelopmental morbidity in early childhood. Acta Paediatr..

