# Supplementary material for: Clinical Significance of Severe and Borderline Umbilical Acidemia: Arterial pH, Lactate, Base Excess and Apgar Score Dynamics in Early Neonatal Risk Stratification
Source: Diagnostics (Basel). 2026 Oct 29;16(21):3562. doi: 10.3390/diagnostics16213562 (PMC13762274; doi:10.3390/diagnostics16213562)
Supplement: Supplementary file 1 [file diagnostics-16-03562-s001.zip › diagnostics-4571862-supplementary.pdf]

Variable	Total cohort, n=1.989
Maternal BMI, kg/m2, median	29.3 (26.1-32.8)
Gestational age at delivery (weeks), median	39.4 (38.6-40.2)
Vaginal delivery, n (%)	1343 (67.6%)
Caesarean delivery, n (%)	568 (28.6%)
Operative vaginal delivery, n (%)	76 (3.8%)
Maternal obesity (BMI ≥30 kg/m2), n (%)	865 (43.6%)
Diabetes, n (%)	129 (6.5%)
Hypertensive disorder, n (%)	128 (6.5%)
Smoking, n (%)	233 (11.8%)
1 minute Apgar score, median	9
5 minute Apgar score, median	10
Arterial pH, median	7.30 (7.24-7.35)
Lactate, mmol/l, median	3.40 (2.20-4.80)
Base excess, mmol/l, median	-3.00 (-5.40 - 0.06)
pH ≤ 7.20, n (%)	299 (15%)
Close observation or NICU admission, n (%)	31 (1.6%)
CPAP requirement, n (%)	12 (0.6%)

Table S1. Baseline maternal, obstetrics and neonatal characteristics of the study cohort. Values are presented as median (Q1-Q3) or n (%).

Parameter	Median	IQR	Min–Max	Shapiro–Wilk W	p-value
pH	7.30	(7.24-7.35)	6.82-7.60	0.976	p<0.001
lactate (mmol/L)	3.4	(2.20-4.80)	0.80-15.00	0.924	p<0.001
base excess (mmol/L)	-3.00	(-5.40- -0.060)	19.60-24.00	0.977	p<0.001

Table S2. Distribution of umbilical arterial acid–base parameters.

Correlation	Spearman's ρ	p-value
pH vs. lactate	-0.608	p<0.001
pH vs. base excess	0.469	p<0.001
lactate vs. base excess	-0.684	p<0.001

Table S3. Association between acid–base parameters in low-pH subgroups.

Parameter vs. Apgar	Spearman's ρ	p-value
pH vs. Apgar 1	0.070	0.227
pH vs. Apgar 5	0.062	0.287
lactate vs. Apgar 1	-0.074	0.202
lactate vs. Apgar 5	-0.078	0.176
BE vs. Apgar 1	0.007	0.906
BE vs. Apgar 5	0.006	0.918

Table S4. Association between acid–base parameters and Apgar scores in the subgroup of acidemia (pH < 7.20).

pH subgroup	Close observation or NICU admission n(%)	CPAP requirement n(%)
severe acidemia (pH <7.10)	8/52 (15.4 %)	6/52 (11.5 %)
borderline acidemia (pH 7.19-7.10)	23/247 (9.3%)	6/247 (2.4 %)
	p= 0.192[×]	p= 0.008[××]

× Pearson's X^2 test

×× Fisher's exact test

Table S5. Severe vs. borderline acidemia: NICU admission and CPAP.

Variable (median)	Standard care	Close observation or NICU admission	p-value[3]
pH	7.15	7.12	0.006
lactate	6.30	8.60	<0.001
BE	-7.70	-9.90	<0.001
metabolic burden score	0	1	<0.001

Table S6. Median of biochemical parameters and metabolic burden score in case of standard care compared with close observation or NICU admission (low-pH subgroup).

Variable (cut-off)	AUC (95% CI)	Sensitivity (95% CI)	Specificity (95% CI)	PPV (95% CI)	NPV (95% CI)	p-value [1]
pH risk (0.045)	0.651 (0.551-0.75)	80.65% (63.72-90.81)	45.90% (40.03-51.88)	14.71% (10.16-20.81)	95.35% (90.22-97.85)	0.003
Lactate (≥8.05 mmol/l)	0.783 (0.681-0.885)	70.97% (53.41-83.90)	84.70% (79.90-88.52)	34.94% (24.33-47.25)	96.19% (92.91-97.98)	<0.001
Base deficit (≥8.95 mmol/l)	0.721 (0.633-0.810)	70.97% (53.41-83.90)	67.54% (61.72-72.86)	20.18% (13.72-28.67)	95.26% (91.24-97.49)	<0.001
Metabolic burden score (≥0.5)	0.759 (0.678-0.840)	77.42% (60.19-88.60)	76.12% (70.67-80.83)	27.27% (19.07-37.38)	96.68% (93.31-98.38)	<0.001
Apgar burden (≥2.5)	0.698 (0.601-0.795)	48.39% (31.97-65.16)	91.04% (87.02-93.91)	38.46% (24.89-54.10)	93.85% (90.24-96.18)	<0.001

Table S7. Optimal ROC-derived thresholds associated with close observation or NICU admission.

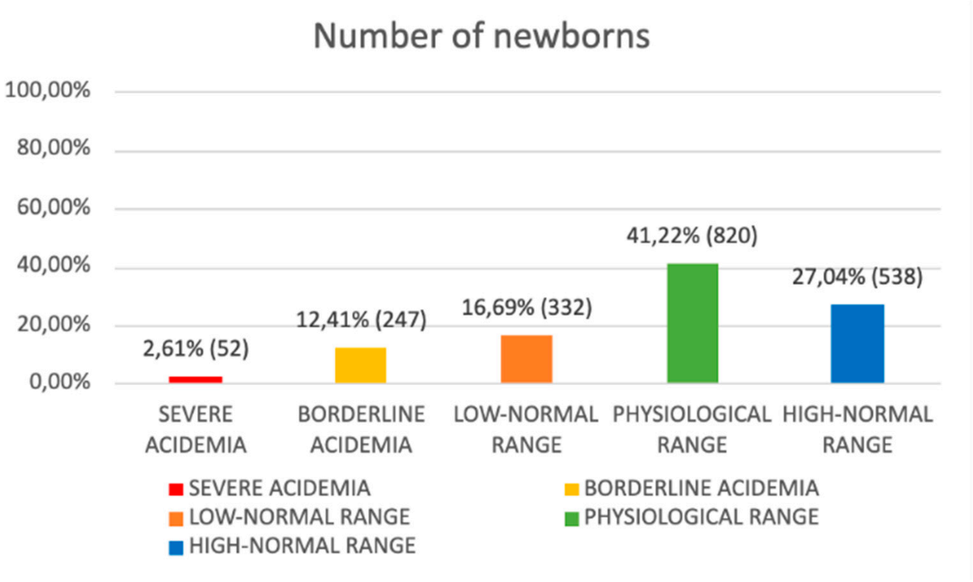

Figure S1. Distribution of umbilical arterial pH subgroups.

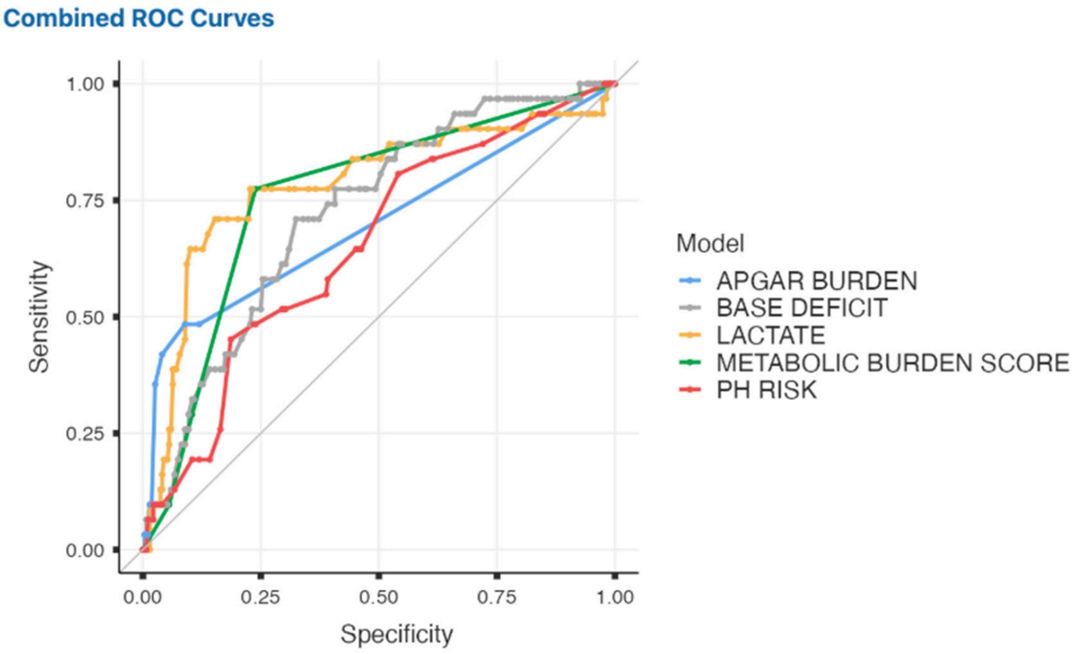

Figure S2. Combined ROC analysis.
